# Using Electronic Referrals to Address Health Disparities and Improve Blood Pressure Control

**DOI:** 10.5888/pcd16.180583

**Published:** 2019-08-22

**Authors:** Amy Bettano, Thomas Land, Alice Byrd, Susan Svencer, Laura Nasuti

**Affiliations:** 1 Massachusetts Department of Public Health, Boston, Massachusetts; 2 Department of Medicine, University of Massachusetts Medical School, Worcester, Massachusetts; 3 National Association of Chronic Disease Directors, Decatur, Georgia

## Abstract

**Introduction:**

Massachusetts developed and used bidirectional electronic referrals to connect clinical patients across the state to interventions run by community organizations. The objective of our study was to determine whether the use of Massachusetts’s electronic referral system (MA e-Referral) reached racial/ethnic groups experiencing health disparities and whether it was associated with improved health outcomes.

**Methods:**

We assembled encounter-level medical records from September 2013 through June 2017 for patients at Massachusetts clinics funded by the Clinical Community Partnerships for Prevention into 2 cohorts. First, all patients meeting program eligibility guidelines for an e-Referral (N = 21,701) were examined to assess the distribution of e-Referrals among populations facing health disparities; second, a subset of 3,817 people with hypertension were analyzed to detect changes in blood pressure after e-Referral to an evidence-based community intervention.

**Results:**

Non-Hispanic black (OR, 1.4; 95% confidence interval [CI], 1.2–1.6) and Hispanic patients (OR, 1.3; 95% CI, 1.1–1.4) had higher odds than non-Hispanic white patients of being referred electronically. Patients completing their hypertension intervention had 74% (95% CI, 1.2–2.5) higher odds of having an in-control blood pressure reading than patients who were not electronically referred.

**Conclusion:**

Clinical to community linkage to interventions through MA e-Referral reached non-Hispanic black, Hispanic, and Spanish-speaking populations and was associated with improved blood pressure control.

**Summary**

**What is already known about this topic?**

Previous electronic referral evaluations focused only on clinical settings and did not examine differences in referral and completion rates by racial/ethnic groupings and preferred language.

**What is added by this report?**

From 2014 through 2017, Massachusetts clinics used electronic referrals to connect underserved patients to community organizations. Non-Hispanic black and Hispanic patients had higher odds than non-Hispanic white patients of receiving a referral, and patients who completed their hypertension intervention had higher odds of controlled blood pressure.

**What are the implications for public health practice?**

This research demonstrates that electronic referral systems can successfully direct referrals to community organizations to address health disparities and improve health outcomes.

## Introduction

Racism, classism, sexism, and socioeconomic inequalities prevent people from achieving good health (1). These conditions create health disparities — the variation in health outcomes between groups that are “systematic, socially produced (and therefore modifiable), and unfair” (2). Massachusetts created the Prevention and Wellness Trust Fund to address health disparities and improve health outcomes. Nine communities covering 15% of the Massachusetts population were granted funding under the fund’s Clinical–Community Partnerships for Prevention (CCPP) (3). These communities had 23% higher rates of hospitalization, greater racial/ethnic diversity, more residents living below the poverty line, and shorter lifespans than the state average (3).

In 2008, Frieden and Mostashari proposed using electronic health records (EHRs) to achieve health improvements (4). Multiple sites and countries previously implemented electronic referrals to link primary care to specialists (5–11). Studies of electronic referrals to tobacco cessation services found increased referral rates, uptake of services, and quit rates relative to other referral methods (12–15). Current research has not examined whether different racial/ethnic populations consent at the same rate to electronic referrals, and research is limited on outcomes outside of tobacco use.

In 2013, Massachusetts secured a State Innovation Model Testing Award from the Centers for Medicare and Medicaid Innovation; from that award Massachusetts developed the first fully electronic, bidirectional referral system between clinicians and community resources (16). The Massachusetts e-Referral system deviates from other electronic referral methods in 2 ways: 1) information moves in both directions between the referring provider and the receiving organization, and 2) the organizations receiving referrals are community-based (16,17). All CCPP partnerships were required to implement an e-referral system. The objective of our study was to determine if clinical sites using the MA e-Referral system successfully reached racial/ethnic groups experiencing health disparities. Additionally, our analyses assessed the efficacy of e-Referrals for community organizations in achieving health improvements.

## Methods

Encounter-level health records on all patients at clinics participating in CCPP were the primary data source. These data are sent quarterly to the Massachusetts Department of Public Health to evaluate quality improvement efforts. Each patient is assigned a random identification number to link records. Information collected includes diagnosis codes, medications, demographics (such as self-reported race/ethnicity and preferred language), laboratory values, and vital signs.

MA e-Referral data were captured in a separate database and matched to the clinical records through an outside agency. Information in the MA e-Referral database covered patients referred, the intervention to which they were referred, and their intervention progress. The linked database resulted in a clinical data set of more than 4 million records for 430,085 patients for visits from September 1, 2013, through June 30, 2017; 99% of electronic referrals were successfully linked to medical records. From this data set, a retrospective cohort analysis was performed on clinical patients in the CCPP. The CCPP program and data were determined to be nonresearch by the Massachusetts Department of Public Health’s institutional review board.

**Figure 1:**
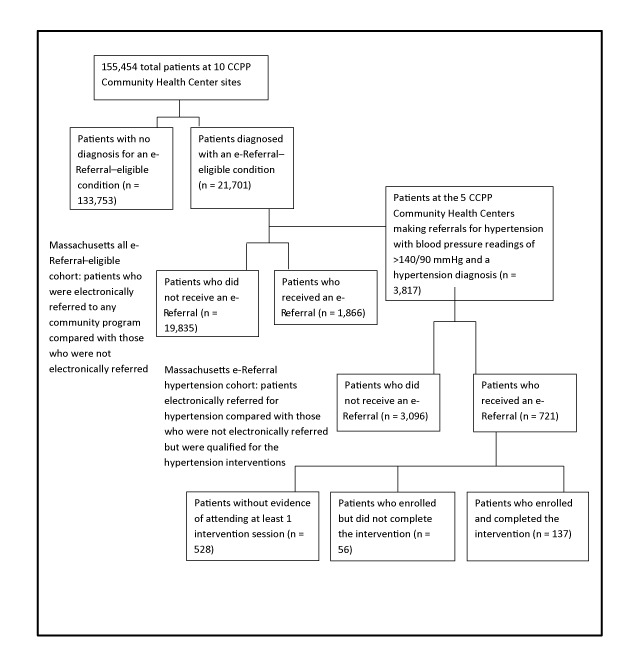
Flowchart of the Massachusetts all e-Referral–eligible cohort and the Massachusetts e-Referral hypertension cohort. The flowchart depicts the progression of the patient population from the 10 Clinical Community Partnerships for Prevention (CCPP) Community Health Center sites to the Massachusetts all e-Referral–eligible cohort (n = 21,701) and the Massachusetts e-Referral hypertension cohort (n = 3,817).

Each CCPP partnership was required to implement an electronic referral system in at least 1 clinical site. Ten health center sites representing 8 of the 9 partnerships (1 partnership invested in its own electronic referral system) implemented MA e-Referral to connect their patients (N = 155,454) to 13 evidence-based community interventions that ranged from self-monitored blood pressure and chronic disease self-management for hypertension to home visits for patients at risk for falls (details of interventions are in the Prevention and Wellness Trust Fund Legislative Report [3]). Health centers selected which conditions they wanted to address in their populations. People who received a diagnosis of a condition eligible for e-Referral (n = 21,701) were separated into those who were electronically referred (n = 1,866) to any community program compared with those not electronically referred (n = 19,835). Multiple logistic regression models were used to analyze differences in referral patterns and completion rates by race/ethnicity and preferred language; 95% confidence intervals were used to establish significance (Figure 1).

The second portion of the analysis focused on determining whether health improvements were associated with the delivery of community-based interventions. For these analyses, only people who had or were eligible for hypertension e-Referrals were examined. Blood pressure readings emerged as a primary focus of this analysis because more patients were referred to community services for hypertension than for any other health condition. Patients at the 5 sites addressing hypertension who met eligibility criteria for a hypertension referral on at least 1 visit in the baseline year were divided into those who were electronically referred versus not electronically referred (n = 3,817 patients in the Massachusetts hypertension cohort sample). The CCPP eligibility criteria for a hypertension referral were having a recorded hypertension diagnosis code during the baseline year and 1 or more blood pressure readings during the baseline year that indicated pressure was not under control (≥140/90 mmHg) per JNC 7 (Joint National Committee on Prevention, Detection, Evaluation, and Treatment of High Blood Pressure–7) guidelines (3). Those receiving hypertension e-Referrals (group 1, n = 721) to either self-monitored blood pressure or chronic disease self-management (3) and those who met the criteria but who were not referred (group 2, n = 3,096) were compared to assess blood pressure changes over time (Figure 1). Of the 721 patients electronically referred, 528 lacked documented evidence in the e-Referral system of attending at least 1 session of the intervention, 56 individuals were enrolled but did not complete the intervention, and 137 individuals enrolled and completed the intervention. The timeframe for these analyses was individualized to each clinical site: baseline data were defined as the year leading up to the date of the clinic’s first e-Referral for hypertension, and intervention data were all post that point. Start dates for hypertension e-Referrals ranged from April through November 2015. The study population was limited to patients with at least 1 follow-up visit during the intervention period.

Sex (male, female, other), self-reported race/ethnicity (non-Hispanic white, non-Hispanic black, Hispanic, other), preferred language for medical information (English, Spanish, other), age group (18–34, 35–54, 55–74, ≥75), and comorbidities (as defined by the Charlson Comorbidity Index [18,19]) were included in the hypertension models as covariates. The outcomes were blood pressure control status and systolic readings based on the last documented blood pressure during the intervention period. Systolic blood pressure was selected because systolic decreases have a greater impact than diastolic decreases on improving cardiac health outcomes (20–22). These blood pressure readings were analyzed with a multiple logistic regression model to detect transition to controlled blood pressure from baseline through the intervention period, and we used a multiple linear regression model to detect millimeters of mercury (mmHg) changes in systolic blood pressure; 95% confidence intervals were used to establish significance. SAS version 9.3 (SAS Institute) was used for all analyses performed.

## Results

### Demographic comparison of referred patients with other groups

Although Massachusetts residents are 80% non-Hispanic white and 16% speak a language other than English as the primary language at home (3), the demographics of the patient population seen at the 10 clinical sites indicated that the CCPP clinics reached a more diverse population. The 155,454 patients were 36.5% non-Hispanic white, and only slightly more than half preferred to have medical information presented in English (56.1%) (Table 1). We performed χ^2^ tests of independence on both the all e-Referral–eligible cohort and the e-Referral hypertension cohort; the distribution of demographics in each sample was related to referral status.

**Table 1:** Characteristics of Patients in the Massachusetts e-Referral Program, September 2013–June 2017^a^

Variable	All Clinical Patients at e-Referral Sites (%)	All e-Referral–Eligible Cohort (%)		e-Referral Hypertension Cohort (%)	
		Not Referred	Referred	Not Referred	Referred
**Total Population**	155,454 (100.0)	19,835 (91.4)	1866 (8.6)	3,096 (81.1)	721 (18.9)
**Race/ethnicity**					
Non-Hispanic black	16,233 (10.4)	2,027 (10.2)	237 (12.7)	379 (12.4)	127 (17.6)
Hispanic	59,000 (38.0)	7,754 (39.1)	918 (49.2)	1,263 (40.8)	390 (54.1)
Non-Hispanic white	56,702 (36.5)	7,375 (37.2)	583 (31.2)	1,096 (35.4)	161 (22.3)
Other **b**	23,519 (15.1)	2,679 (13.5)	128 (6.9)	358 (11.6)	43 (6.0)
**Sex**					
Male	68,572 (44.1)	8,937 (45.1)	715 (38.3)	1,599 (51.7)	432 (59.9)
Female	86,874 (55.9)	10,897 (54.9)	1,151 (61.7)	1,497 (48.4)	289 (40.1)
Other	8 (0.0)	1 (0.0)	0 (0.0)	0 (0.0)	0 (0.0)
**Preferred language^c^**					
English	87,218 (56.1)	9,476 (47.8)	878 (47.1)	1,445 (46.7)	354 (49.1)
Spanish	41,495 (26.7)	6,290 (31.7)	783 (42.0)	1,063 (34.3)	338 (46.9)
Other	26,741 (17.2)	4,069 (20.5)	205 (11.0)	588 (19.0)	29 (4.0)
**≥1 Charlson comorbidities present^d^**	34,310 (22.1)	10,465 (52.8)	1,397 (74.9)	2,355 (76.1)	553 (76.7)
**Age**					
0–17	36,378 (23.4)	1,140 (5.8)	77 (4.1)	0 (0.0)	0 (0.0)
18–34	42,143 (27.1)	1,007 (5.1)	121 (6.5)	72 (2.3)	17 (2.4)
35–54	45,757 (29.4)	4,763 (24.0)	504 (27.0)	1,017 (32.9)	245 (34.0)
55–74	27,024 (17.4)	10,032 (50.6)	917 (49.1)	1,675 (54.1)	423 (58.7)
≥75	4,152 (2.7)	2,893 (14.6)	247 (13.2)	332 (10.7)	36 (5.0)

a Percentages may not total 100 because of rounding.

b Includes all patients not classified as non-Hispanic black, Hispanic, or non-Hispanic white.

c The primary language that the patient selects to receive medical information.

d Charlson comorbidity index (18,19).

**Figure 2:**
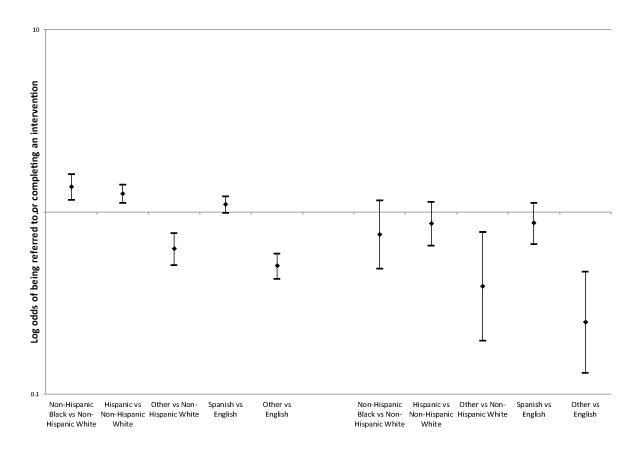
Multiple logistic regression modeling of the odds of receiving and completing an e-Referral by race/ethnicity and preferred language among 21,701 Massachusetts clinical patients seen from 2013 through 2017. The multiple logistic regression models examine the odds of referral and the odds of completing an intervention by race/ethnicity and preferred language. Brackets indicate 95% Wald confidence intervals. Abbreviation: NH, non-Hispanic.

E-Referrals were first made in 2014. From 2014 through 2017, 1,866 patients received at least one e-Referral. All race/ethnicity and language referral analyses controlled for age category, sex, and Charlson comorbidity presence. Among these referrals, Hispanic patients had 26% higher odds of being electronically referred than non-Hispanic white patients (95% CI, 1.1–1.4) and non-Hispanic black patients had 37% higher odds (95% CI, 1.2–1.6) (Figure 2).

**Table:** 

Variable	Odds Ratio (95% Confidence Interval)
**Odds of electronic referral**	
Non-Hispanic black vs non-Hispanic white	1.4 (1.2–1.6)
Hispanic vs non-Hispanic white	1.3 (1.1–1.4)
Other vs non-Hispanic white	0.6 (0.5–0.8)
Spanish vs English	1.1 (1.0–1.2)
Other vs English	0.5 (0.4–0.6)
**Odds of completing intervention**	
Non-Hispanic black vs non-Hispanic white	0.8 (0.5–1.2)
Hispanic vs non-Hispanic white	0.9 (0.7–1.1)
Other vs non-Hispanic white	0.4 (0.2–0.8)
Spanish vs English	0.9 (0.7–1.1)
Other vs English	0.3 (0.1–0.5)

Next, completion rates were examined with logistic regression models of race/ethnicity and preferred language that controlled for age category, sex, and Charlson comorbidity status to determine whether all groups had the same odds of completing interventions once they were electronically referred. Completion rates among Hispanic patients (OR 0.9; 95% CI, 0.7–1.1) and non-Hispanic black patients (OR 0.8; 95% CI, 0.5–1.2) were not significantly different from non-Hispanic white patients’ completion rates. By language, completion rates among Spanish speakers and English speakers were also not significantly different (OR 0.9; 95% CI, 0.7–1.1) (Figure 2).

### Hypertension health outcomes of referred patients compared with nonreferred patients

A multiple logistic regression model was used to calculate the odds that an individual maintained or achieved blood pressure control (<140/90 mmHg) at their last reading during the intervention time period when compared with their last reading during baseline. During the last visit of the baseline period, 62% of the nonreferred group had hypertensive blood pressure compared with 58% of the electronically referred group. After controlling for sex, presence of comorbidities, age group, race/ethnicity, and preferred language, patients who were electronically referred (regardless of the referral outcome) had 66% greater odds (95% CI, 1.4–2.0) of maintaining or achieving blood pressure control when compared with nonreferred patients. Patients who were referred and completed their program had 74% greater odds (95% CI, 1.2–2.5) than nonreferred patients of moving into or maintaining blood pressure control (Table 2). At the end of the intervention period, 43% of the nonreferred group had hypertensive blood pressure compared with 33% of the electronically referred group (*P* < .01). When controlling for referral and completion status, non-Hispanic black patients had lower odds of achieving blood pressure control than non-Hispanic white patients, whereas Hispanic patients and Spanish-speaking patients did not have significantly different rates of control achievement than non-Hispanic white patients and English-speaking patients (Table 2). All groups saw a decrease in the percentage of their population with hypertensive blood pressure readings, but only for Hispanic and non-Hispanic white patients was the difference in the referred group significantly lower than in the nonreferred group (*P* < .01 for both).

**Table 2:** Comparison of 2 Models for Odds of Blood Pressure Control During the Intervention, Patients in Massachusetts e-Referral Program, September 2013–June 2017^a^

Variable	Model 1: All Massachusetts e-Referral for Hypertension Patients and Patients Not e-Referred (n = 3,817), OR (95% CI)^a^	Model 2: Patients Completing Massachusetts e-Referral for Hypertension and Patients Not e-Referred (n = 3,233), OR (95% CI)^a^
**Race/ethnicity**		
Non-Hispanic white	Reference	
Non-Hispanic black	0.7 (0.6–0.9)	0.8 (0.6–1.0)
Hispanic	0.8 (0.6–1.0)	0.9 (0.7–1.2)
Other **b**	1.0 (0.8–1.3)	1.0 (0.8–1.4)
**Age, y**		
18–34	Reference	
35–54	0.7 (0.4–1.0)	0.6 (0.4–1.0)
55–74	0.8 (0.5–1.2)	0.7 (0.4–1.2)
≥75	0.8 (0.5–1.3)	0.7 (0.4–1.3)
**Preferred language^c^**		
English	Reference	
Other	1.1 (0.9–1.4)	1.1 (0.9–1.4)
Spanish	1.1 (0.9–1.4)	1 (0.8–1.3)
**Sex**		
Male	Reference	
Female	1.1 (1.0–1.3)	1.1 (1.0–1.3)
**Comorbidity^d^**		
Absent	Reference	
Present	1.3 (1.1–1.5)	1.3 (1.1–1.6)
**Referral status^e^**		
No referral	Reference	
Referred	1.7 (1.4–2.0)	—f
Completed referral	—f	1.7 (1.2–2.5)

Abbreviations: CI, confidence interval; OR, odds ratio.

a Multiple logistic regression model of the odds ratios for last blood pressure transitioning to or remaining in control when compared with baseline blood pressure in the Massachusetts e-Referral hypertension cohort population. Values are odds ratio and 95% confidence intervals for in-control blood pressure. Wald 95% confidence intervals were used to establish significance.

b Includes all patients not classified as non-Hispanic black, Hispanic, or non-Hispanic white.

c The primary language that the patient selects to receive medical information.

d Defined as the presence of hypertension and 1 or more of the conditions covered by the Charlson comorbidity index (18,19).

e Defined as whether patients received an e-Referral and whether they completed that e-Referral.

f Outcome not applicable for that logistic regression model.

To determine the extent of changes in systolic blood pressure readings, the final systolic readings were compared among the groups in a linear regression. During the last visit of the baseline period, the nonreferred group had an average systolic blood pressure reading of 139 mmHg, compared with 138 mmHg in the electronically referred group. After controlling for sex, Charlson comorbidities, age group, race/ethnicity, and preferred language, patients who were referred (regardless of the referral outcome) had a final systolic blood pressure reading that was on average 3.4 mmHg lower (*P* < .01) than patients without a referral. When limiting the referral sample to only completers, referred patients who completed their intervention had a final systolic blood pressure reading that was on average 3.0 mmHg lower (*P* = .04) than patients without a referral (Table 3). At the end of the intervention period, the nonreferred group had an average systolic blood pressure reading of 134 mmHg compared with 131 mmHg in the electronically referred group. Non-Hispanic black patients had a higher final systolic blood pressure than non-Hispanic white patients. Hispanic patients did not have significantly different systolic blood pressure readings than non-Hispanic white patients, nor did Spanish-speaking patients have significantly different systolic blood pressure readings than English-speaking patients (Table 3). All racial/ethnic referred groups, except for non-Hispanic white patients, saw an average decrease in their systolic blood pressure readings that was more than double what was observed in their nonreferred counterparts.

**Table 3:** Comparison of Systolic Blood Pressure Measurements During the Intervention, Calculated by Multiple Linear Regression, Patients in Massachusetts e-Referral Program, April 2015–June 2017

Characteristic	Model 1: All MA e-Referred for Hypertension Patients and Not e-Referred (n = 3,817)		Model 2: Only Completing MA e-Referral for Hypertension Patients and Not-e-Referred (n = 3,233)	
	Mean Change in Systolic Blood Pressure (mmHg)	*P* Value^a^	Mean Change in Systolic Blood Pressure (mmHg)	*P* Value^a^
**Race/ethnicity**				
Non-Hispanic white	Reference			
Non-Hispanic black	3.2	<.001	3.0	.001
Hispanic	0.9	.38	−0.3	.77
Other **b**	−0.07	.94	0.4	.73
**Age**				
18–34	Reference			
35–54	2.9	.1	3.7	.06
55–74	4.6	.01	5.2	.01
≥75	5.2	.01	5.5	.01
**Preferred language^c^**				
English	Reference			
Other	0.3	.72	0.06	.94
Spanish	−0.3	.73	0.7	.52
**Sex**				
Male	Reference			
Female	−1.5	.01	−1.0	.09
**Comorbidity^d^**				
Absent	Reference			
Present	−1.8	.005	−2.0	.004
**Referral status^e^**				
No referral	Reference			
Referred	−3.4	<.001	—f	—f
Completed referral	—f	—f	−3.0	.04

a *P* values calculated by using *t* test; α level for significance was *P* < .05.

b Includes all patients not classified as non-Hispanic black, Hispanic, or non-Hispanic white.

c The primary language that the patient selects to receive medical information.

d Defined as the presence of hypertension and 1 or more of the conditions covered by the Charlson comorbidity index (18,19).

e Defined as whether patients received an e-Referral and whether they completed that e-Referral.

f Outcome not applicable for that linear regression model.

## Discussion

Electronic referrals have been found to better ensure the delivery of referrals, improve documentation, and standardize the referral format when compared with other referral methods (5–9). Embedding referral information in medical records provided the opportunity to assess the efficacy of MA e-Referral as a bidirectional referral system establishing clinical to community linkages (7). Moreover, MA e-Referral allowed Massachusetts to assess whether referrals were being directed toward groups experiencing health disparities. Nationally, hypertension is a prominent health equity issue. Rates among non-Hispanic black people in the United States are among the highest worldwide, and this population develops high blood pressure at younger ages than non-Hispanic white populations and has higher average blood pressure levels (23), which persist even after controlling for socioeconomic status and underlying medical conditions (24,25). Our analysis controlled for the presence of comorbidities, which are correlated with hypertension rates and cluster in populations to produce some of the hypertension health disparities (26). People with 1 or more comorbidities were 30% more likely to have in-control blood pressure.

Promisingly, our analysis found that referrals were successfully focused on groups facing hypertension health disparities: among eligible candidates, non-Hispanic black and Hispanic patients had higher odds of receiving an e-Referral than non-Hispanic white patients. The MA e-Referral system allowed for examination of completion rates by race/ethnicity to determine whether e-Referrals led to participation in community programs. Completion rates by race/ethnicity and language were not significantly different when compared with non-Hispanic white patients or with English-speaking patients’ completion rates.

Both receiving and completing an e-Referral for a community hypertension intervention were found to be correlated with increased likelihood of achieving blood pressure control and reducing systolic blood pressure. The 3.0 mmHg reduction in systolic blood pressure that referral completers experienced is on par with the reductions seen in many nonpharmacologic interventions promoted by the 2017 High Blood Pressure Clinical Practice Guidelines (26).

This research had some limitations. First, although all clinical sites received guidelines on how to refer patients, there was flexibility in guideline application. If patients did not conform to referral criteria, but clinical judgment determined that the program would be valuable, a referral could be made. Data indicate the guidelines were typically observed, because 74% of hypertension e-Referral patients met JNC 7 guidelines before referral. The nonreferred group, all of whom met JNC 7 guidelines, may not accurately represent the health status of the electronically referred patients (in the absence of a referral). Furthermore, the offer of an e-Referral to patients was not documented in medical records. Because accepting an e-Referral intervention was voluntary, participating patients may have been more motivated to make behavior changes to reduce their hypertension risk beyond intervention participation, potentially introducing self-selection bias (28). Additionally, our clinics are relatively homogenous in serving low-income populations that were racially, ethnically, and linguistically diverse. The linear modeling outlined in this article was also run as a mixed-effects model with clinic as a random effect; clinical effect was not significant. Though it did not affect the model, the unique make-up of our clinical populations may limit generalizability of the results. Lastly, the MA e-Referral system was designed to facilitate communication between clinicians and community service providers rather than as a data collection system for research. Consequently, we had issues with the standardization of the MA e-Referral data for the term “enrollment.” All e-Referrals were reviewed by the first author to systematize the classification of enrollment as at least 1 session attended. Not all sites were able to implement e-Referral because of technology or budget limitations and instead made referrals via paper copies and fax. These referrals were not used in this analysis. However, data collected on all methods of hypertension referrals to community interventions established the enrollment rate at approximately 50% (3). Even after reclassification, this analysis found only a 27% referral enrollment rate. It is unclear if more patients enrolled than were able to be identified on the basis of the documentation in the e-Referral system. Future analyses of the benefits of enrolling (even without completion) would expand the evidence base around the effectiveness of these interventions.

Patients completing interventions to which they were referred had higher odds of transitioning to an in-control blood pressure status and of experiencing reductions in their overall systolic blood pressure when compared with nonreferred patients with similar demographic and health profiles. Additionally, e-Referrals were successfully directed toward groups facing health disparities, and completion rates were not statistically different between groups after the e-Referral was made. These findings demonstrate the successful implementation of the MA e-Referral system as a means to connect patients to evidence-based interventions to improve hypertension outcomes and of the ability to direct referrals to address health disparities, both critical steps toward improved population health.
